# Mining key genes associated with phosphorus deficiency through genome-wide identification and characterization of cucumber *SPX* family genes

**DOI:** 10.1186/s12870-024-05436-3

**Published:** 2024-07-24

**Authors:** Jialin Li, Linyue Hu, Qianqian Luan, Jingdan Zhang, Xueru Feng, Hongmei Li, Zenghui Wang, Wenxing He

**Affiliations:** 1 School of Biological Science and Technology, University of Jinan, Jinan, 250022 China; 2 Shandong Institute of Pomology, Tai’an, Shandong 271000 China; 3 Gansu Agricultural Engineering Technology Research Institute, Lanzhou, 730000 China

**Keywords:** Cucumber, *SPX* family, Pi starvation, PHR1, Regulatory networks

## Abstract

**Background:**

Proteins harboring the SPX domain are crucial for the regulation of phosphate (Pi) homeostasis in plants. This study aimed to identify and analyze the entire *SPX* gene family within the cucumber genome.

**Results:**

The cucumber genome encompassed 16 *SPX* domain-containing genes, which were distributed across six chromosomes and categorized into four distinct subfamilies: *SPX*, *SPX-MFS*, *SPX-EXS* and *SPX-RING*, based on their structure characteristics. Additionally, gene duplications and synteny analysis were conducted for *CsSPXs*, revealing that their promoter regions were enriched with a variety of hormone-responsive, biotic/abiotic stress and typical P1BS-related elements. Tissue expression profiling of *CsSPX* genes revealed that certain members were specifically expressed in particular organs, suggesting essential roles in cucumber growth and development. Under low Pi stress, *CsSPX1* and *CsSPX2* exhibited a particularly strong response to Pi starvation. It was observed that the cucumber cultivar Xintaimici displayed greater tolerance to low Pi compared to black-spined cucumber under low Pi stress conditions. Protein interaction networks for the 16 CsSPX proteins were predicted, and yeast two-hybrid assay revealed that CsPHR1 interacted with CsSPX2, CsSPX3, CsSPX4 and CsSPX5, implying their involvement in the Pi signaling pathway in conjunction with CsPHR1.

**Conclusion:**

This research lays the foundation for further exploration of the function of the *CsSPX* genes in response to low Pi stress and for elucidating the underlying mechanism.

**Supplementary Information:**

The online version contains supplementary material available at 10.1186/s12870-024-05436-3.

## Introduction

Phosphorus (P) is a fundamental element for the growth and development of plants, required in considerable quantities [1]. It is implicated in a multitude of metabolic pathways, playing a pivotal role not only in the structure of biofilms and nucleic acids, but also in essential processes such as photosynthesis, modulation of enzyme activities, respiration, signal transduction, energy metabolism, and carbon assimilation [2–4]. Additionally, phosphorus plays an essential role in enhancing plant resistance. For instance, under low-temperature stress, phosphorus enhances the levels of phospholipids and soluble sugars in plants, thereby sustaining normal physiological functions. A deficiency in phosphorus can severely compromise the growth and development as well as yield of different crops [5–8]. Given the dynamic fluctuations of phosphorus levels in the surrounding environment, cellular mechanisms must engage to preserve internal phosphorus homeostasis, ensuring optimal survival. To achieve this balance, organisms must detect variations in phosphorus concentrations both within and outside the system and initiate adaptive measures. These include modulating gene expression, metabolism, and tissue morphology to acclimate to phosphorus deficiency [9–12]. Recent studies have identified numerous genes at the molecular level that are involved in the plant’s response to low Pi levels, collectively constituting the phosphorus signaling regulatory pathway in plants. Proteins containing the SPX domain are widely involved in the regulation of phosphorus signaling networks [13, 14], and the name SPX domain is derived from the acronyms of the three proteins in which it was first identified: the yeast SYG1 protein [15], the PHO81 protein [16], and the human XPR1 protein [17]. Typically, the SPX domain is located at the N-terminus of proteins and consists of approximately 180 amino acids. In yeast, the SPX domains of Pho87 and Pho90 regulate the activity of Pi uptake and influence the regulation of the Pi signaling pathway [18]. In plants, SPX domain-containing proteins are essential for Pi homeostasis and signaling transduction [19], and the *SPX* family genes are divided into four subfamilies based on the presence of other conserved domains. Among them, proteins containing only the SPX domain are referred to as the *SPX* subfamily [20]; those with an additional MFS (Major Facility Superfamily) domain at the C-terminus, in addition to the N-terminal SPX domain are denoted as the *SPX-MFS* subfamily; the *SPX-RING* subfamily also contains the RING (Really Interesting New Gene) domain at the C-terminus; and the *SPX-EXS* subfamily contains the EXS (ERD1/XPR1/SYG1) domain at the C-terminus [11]. Specific roles in Pi homeostasis and signaling have been ascribed to several of these genes [21, 22]. In *Arabidopsis* and rice, *SPX1* and *SPX2* have been demonstrated to negatively regulate the phosphorus starvation response by inhibiting PHR1 or PHR2 activity [23, 24]. With the exception of *SPX4*, the remaining *SPX* subfamily genes are induced in both aerial parts and roots in response to phosphorus deficiency [20]. The *SPX-EXS* subfamily, also recognized as PHO1 in eukaryotes, is predominantly expressed in the protoxylem cells of plant roots and is responsible for loading the phosphorus absorbed by the roots into the xylem for transport to aerial parts [25]. The *Atpho1* mutants exhibit increased phosphorus concentration in the root, decreased the phosphorus concentration in the stem, and delayed the development of aerial parts [26, 27]. Overexpression of *OsSPX-MFS3*, a low-affinity phosphorus transporter that facilitates the efflux of phosphorus from the vacuole to the cytosol in conjunction with proton movement, results in a reduced concentration of Pi in rice tissue vacuoles [28]. In transgenic *Arabidopsis*, overexpression of the *CoSPX-MFS3* gene can improve tolerance to low Pi by increasing biomass and organic acid content [21]. Two members of the *SPX-RING* family, also known as *NLA*, have been identified in both *Arabidopsis* and rice, with both being linked to the Pi response and involved in Pi homeostasis [29, 30].

The systematic categorisation of gene family members within the genome constitutes an initial step in exploring biological queries related to species characteristics, and establishes a solid evidence base for subsequent studies of gene function and genetic transformation. Currently, *SPX* family genes have been extensively studied in *Arabidopsis*, rice, rape, soybean, maize and wheat, with 20 *AtSPXs*, 15 *OsSPXs*, 69 *BnaSPXs*, 9 *GmSPXs*, 33 *ZmSPX* and 46 *TaSPXs* identified [11, 14, 20, 31–33]. However, a comprehensive systematic analysis of the *SPX* gene family in cucumber has not been conducted, and the expression patterns of *SPX* genes in different cucumber cultivars under low phosphorus remain unreported. In this study, we identified a total of 16 *CsSPX* genes and performed a suite of bioinformatics analyses for these family members, including gene structures, conserved domains, phylogenetic relationships, synteny analysis, *cis*-acting elements, and protein interaction network maps. Furthermore, we also demonstrated the interactions between CsSPX proteins and CsPHR1. This study provided an initial foray into the molecular functions of the *CsSPX* gene family and will lay the foundation for in-depth biological exploration of this pivotal gene family in cucumber.

## Results

### Identification of *SPX* genes in cucumber

**Fig. 1:**
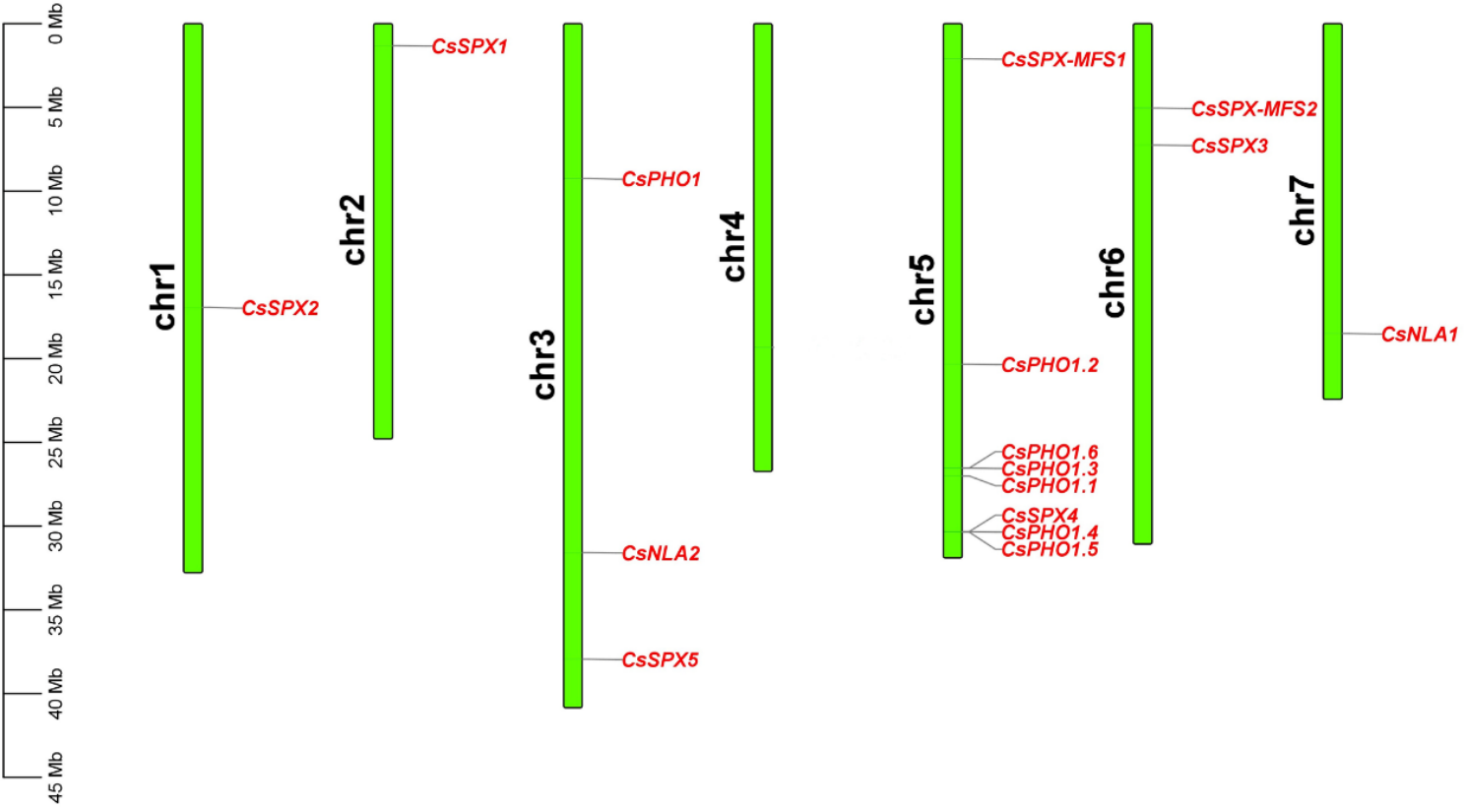
Distribution of *CsSPX* family genes on cucumber chromosomes

Employing a BlastP search within the cucumber genome database, we identified 16 *CsSPX* genes utilizing 20 *Arabidopsis* SPX proteins as the reference sequences. The authenticity of these cucumber *SPX* genes was corroborated using the Pfam database (http://pfam.janelia.org/) and SMART website (http://smart.embl-heidelberg.de/) [34]. Based on their homologs in *Arabidopsis*, the *CsSPX* family genes were designated as follows: five genes were classified as members of the *SPX* subfamily, seven as *SPX*-*EXS* subfamily members, two as *SPX*-*MFS* subfamily members, and two as *SPX*-*RING* subfamily members. Table 1 summarised the gene ID, molecular weight, amino acid length, and isoelectric points (pI) for the 16 *CsSPX* genes. The amino acid sequences of the 16 CsSPX proteins ranged from 246 aa (CsSPX3) to 830 aa (CsPHO1.3), with predicted molecular weights spanning 28.58 kD to 95.80 kD. All members of the *SPX* and *SPX*-*MFS* subfamilies exhibited a pI below 7, denoting their acidic nature, while all members of the *SPX*-*EXS* and *SPX*-*RING* subfamilies, except *CsNLA2*, presented a pI above 7, suggesting a basic character (Table 1). Figure 1 illustrated the chromosomal distribution of *CsSPX* family genes, with 16 *SPX* genes from cucumber effectively mapped onto six chromosomes. Only one gene was present on chromosomes 1, 2 and 7, while chromosome 6 had two genes. Chromosome 3 contained three genes and chromosome 5 had the highest number of genes - eight genes (Fig. 1).

**Table 1:** Description of cucumber SPX family genes

Gene ID		Gene name	Location	Molecular weight (kD)	Amino acid length (aa)	pI
*CsaV3_2G002890*	*CsSPX1*		Chr2: 1309249–1313288	33.39	290	5.13
*CsaV3_1G030190*	*CsSPX2*		Chr1: 16941555–16944650	32.56	286	6.19
*CsaV3_6G009000*	*CsSPX3*		Chr6: 7249890–7252124	28.58	246	6.54
*CsaV3_5G038190*	*CsSPX4*		Chr5: 30321807–30327195	37.00	325	4.97
*CsaV3_3G046460*	*CsSPX5*		Chr3: 37933215–37937774	30.61	263	5.24
*CsaV3_3G011910*	*CsPHO1*		Chr3: 9232360–9241503	88.78	767	9.39
*CsaV3_5G033820*	*CsPHO1.1*		Chr5: 27001449–27006958	95.80	825	9.30
*CsaV3_5G025280*	*CsPHO1.2*		Chr5: 20322929–20327860	92.16	800	9.31
*CsaV3_5G033010*	*CsPHO1.3*		Chr5: 26530988–26536992	95.33	830	9.11
*CsaV3_5G038200*	*CsPHO1.4*		Chr5: 30327522–30331226	90.77	785	9.11
*CsaV3_5G038220*	*CsPHO1.5*		Chr5: 30338753–30344652	91.01	784	9.34
*CsaV3_5G032990*	*CsPHO1.6*		Chr5: 26522669–26527656	70.20	609	8.51
*CsaV3_5G003300*	*CsSPX-MFS1*		Chr5: 2084005–2092264	77.99	696	6.16
*CsaV3_6G005890*	*CsSPX-MFS2*		Chr6: 5022224–5033149	77.49	694	6.68
*CsaV3_7G029190*	*CsNLA1*		Chr7: 18489593–18493659	35.73	313	8.95
*CsaV3_3G038390*	*CsNLA2*		Chr3: 31576664–31580636	33.93	297	6.77

### Analysis of conserved motifs and gene structures in *SPX* gene family

**Fig. 2:**
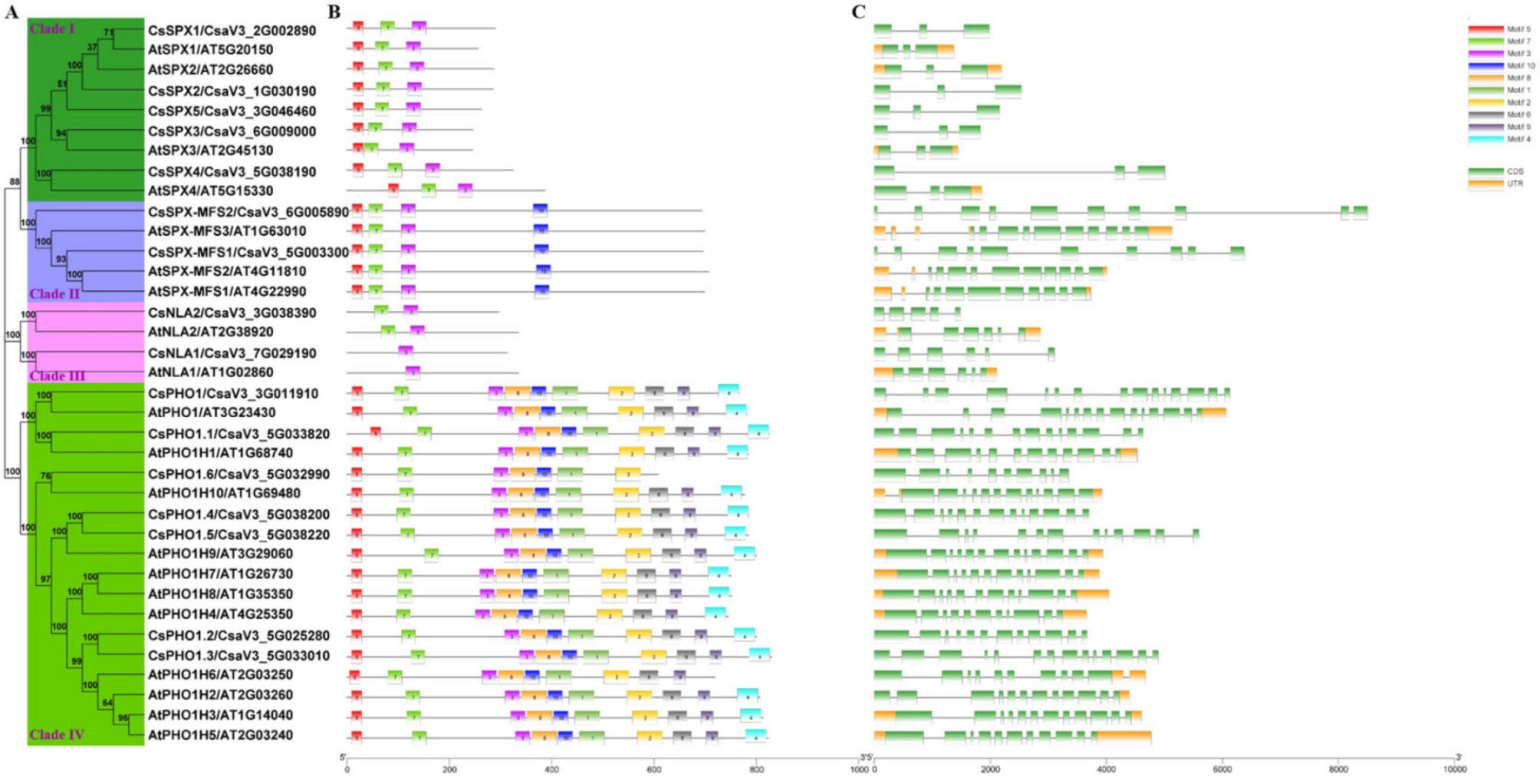
Phylogenetic and structural analyses of *SPX* genes in cucumber and *Arabidopsis*. (**A**) A phylogenetic tree was constructed using MEGA 7.0, containing 16 CsSPX and 20 AtSPX protein sequences, which were grouped into four distinct clades. (**B**) Conserved motifs in SPX proteins were predicted using MEME software, with the 10 identified motifs depicted in Fig. S1. (**C**) The gene structure of *SPXs* was illustrated, providing insights into their exon-intron organization

To establish preliminary evolutionary relationships of cucumber SPX proteins, we conducted a detailed comparative analysis of phylogenetic trees, conserved motifs, and gene structures of 16 CsSPX and 20 AtSPX proteins (Fig. 2). Figure 2A presented a phylogenetic categorization of the 36 SPX proteins into quartets of evolutionary clusters - Clade I, Clade II, Clade III, and Clade IV. These clades were aligned with the *SPX*, *SPX*-*MFS*, *SPX*-*RING*, and *SPX*-*EXS* subfamilies, respectively. Each clade was composed of both *CsSPX* and *AtSPX* genes, with members within each subfamily exhibiting a high degree of evolutionary conservation and sequence homology.

To gain insights into the evolution and function of gene family members, a scrutiny of their gene architectures is essential. The gene structures of the *CsSPXs* were visualized using the Gene Structure Display Server (GSDS), revealing a clear pattern of structural homology within each clade (Fig. 2C). Similar gene structure patterns were observed within the same clade. The *SPX* subfamily members, out of the 36 *SPX* genes, possessed only three exons. In contrast, the *SPX-MFS* subfamily exhibited a more intricate structure, with the number of exons ranging from nine to ten. The *SPX-RING* subfamily consisted of five to six exons, and the *SPX-EXS* subfamily contained ten to fifteen exons (Fig. 2C). This variation in gene architecture across subfamilies might reflect divergent functional specializations and evolutionary adaptations.

To enhance our understanding of the structural diversity of SPX proteins and predict their roles, we utilized MEME for predicting the number and composition of conserved motifs in AtSPX and CsSPX proteins (Fig. 2B). Ten distinct motifs were identified (Fig. S1), with proteins within the same clade exhibiting analogous motif distribution patterns. All SPX proteins contained Motif 3, indicating that this motif was specific to *SPX* family genes and might be related to their shared function. Certain motifs, namely 1, 2, 4, 6, 8, and 9, were exclusively present in all members of Clade IV subfamily and not in other Clades. This observation further validated the accuracy of the subfamily classification. Clade IV possessed the most motifs, totaling 10, while the other three subfamilies had only 1–3 motifs in total (Fig. 2B). Therefore, the roles of these motifs warranted further investigation to comprehend the functioning of these proteins.

In general, closely related *SPX* genes in the phylogenetic tree exhibited similar conserved motifs and gene structures, suggesting that proteins within the same subfamily might share similar functions.

### Analysis of the evolutionary relationships of SPX proteins across various plant species

**Fig. 3:**
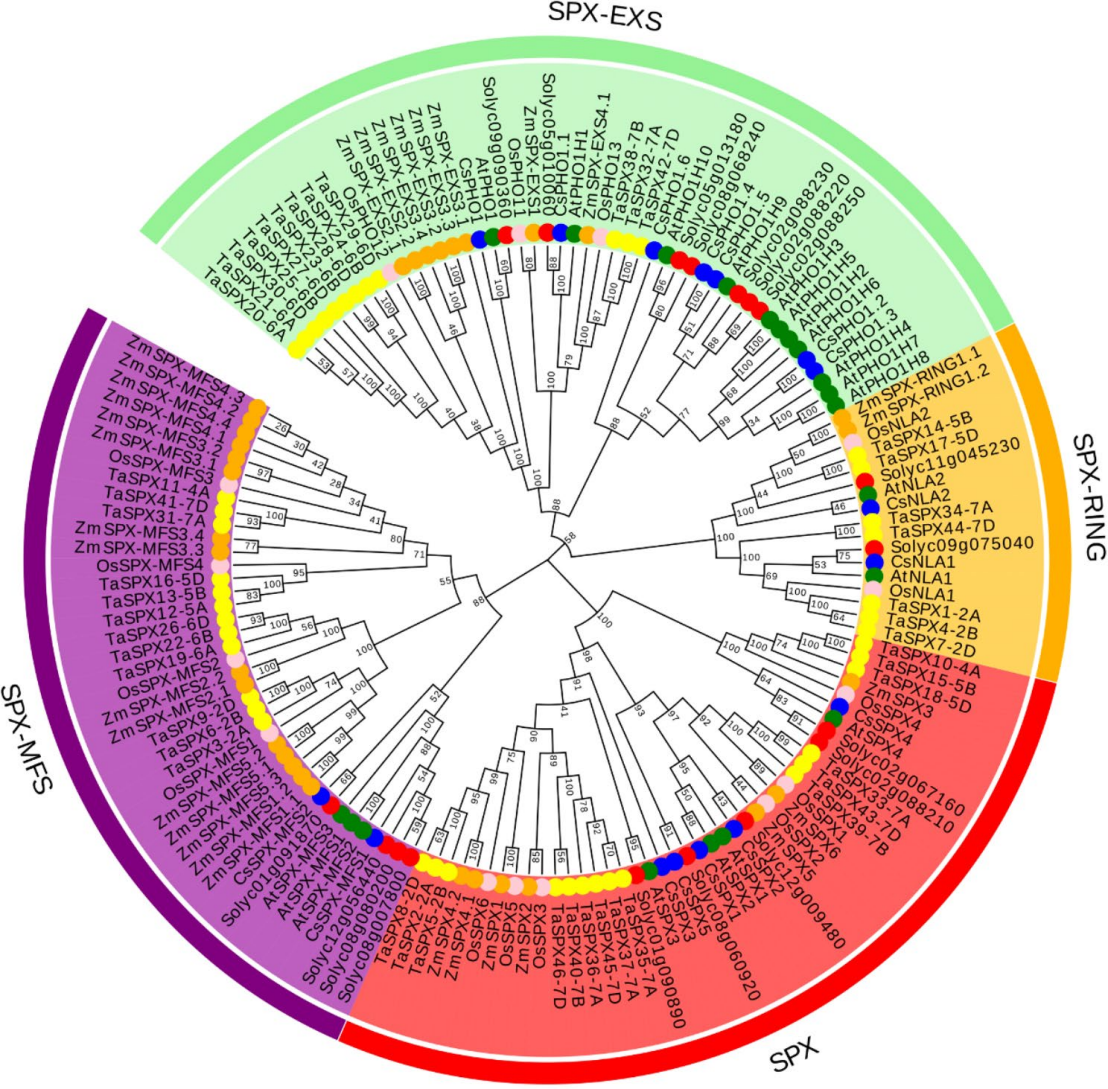
Phylogenetic tree composed of 147 SPX proteins belonging to *Cucumis sativus*, *Solanum lycopersicum*, *Arabidopsis thaliana*, *Triticum aestivum*, *Oryza sativa*, and *Zea mays*. Colour-coded stars indicated SPX proteins from distinct species

To further evaluate the evolutionary relationships among the *SPX* gene family in multiple plant species, we aligned 147 SPX protein sequences using CLUSTALW, encompassing 16 CsSPX, 18 SlSPX, 15 OsSPX, 33 ZmSPX, 46 TaSPX, and 20 AtSPX from six distinct species. Subsequently, we employed the Neighbor-Joining (NJ) method implemented in MEGA 7 to construct a phylogenetic tree, which resulted in the division of proteins into four subfamilies, with SPX-EXS subfamily containing the highest number of proteins (Fig. 3; Table S1). Distinct evolutionary clades were apparent for monocotyledonous species (*Oryza sativa*, *Zea mays*, and *Triticum aestivum*) and dicotyledonous species (*Arabidopsis*, *Cucumis sativus*, and *Solanum lycopersicum*). The members of *SPX*-*EXS* and *SPX*-*MFS* subfamilies exhibited evident species differentiation, indicating that SPX-EXS and SPX-MFS proteins might have varying biological functions in divergent plant species. The observed phylogenetic divergence between monocots and dicots was likely the result of a combination of factors, including functional differentiation, environmental adaptation, genomic divergence, and developmental regulation. These differences highlighted the complexity of plant evolution and the intricate ways in which plants have adapted to their environments over time. Furthermore, we separately compared the evolutionary relationship of *SPX* gene family in *Cucumis sativus* and *Cucurbita moschata*, both within the same family Cucurbitaceae. The findings indicated that genes from the same genera might exhibit a closer phylogenetic relationship (Fig. S2). Overall, a phylogenetic analysis could offer valuable insights into the evolution and function of family genes.

### Synteny analysis of *SPX* genes in tomato, *Arabidopsis* and cucumber

**Fig. 4:**
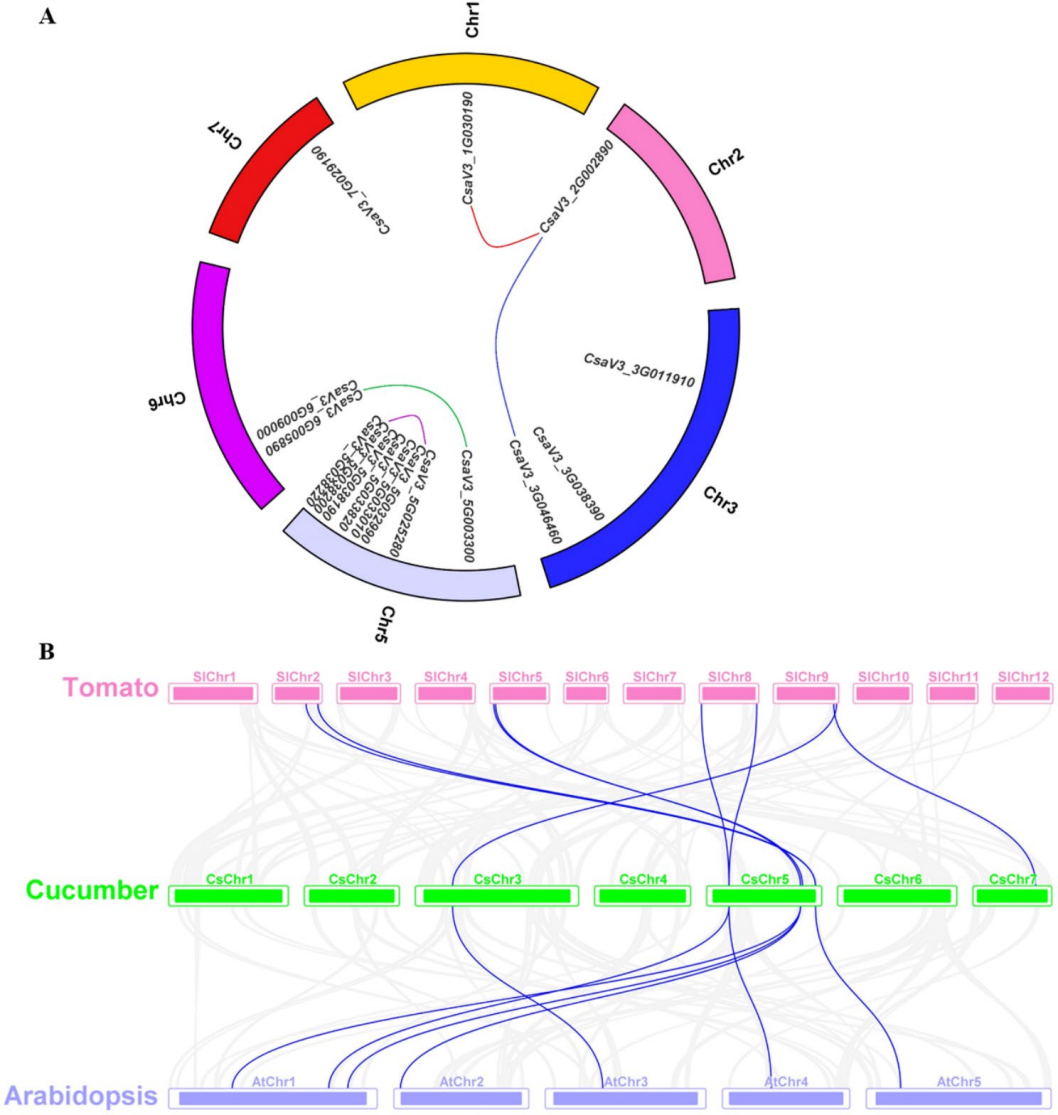
Duplication and synteny analysis of *CsSPX* genes. (**A**) *CsSPX* gene duplication events between chromosomes. Different colored lines connected distinct segmental duplication gene pairs. (**B**) Synteny analysis of *SPX* genes between cucumber and *Arabidopsis* and tomato revealed collinear blocks of the *SPX* gene within the three genomes, as denoted by the blue lines

Gene duplication is a significant driving force in genome and genetic system evolution [35]. The expansion of plant gene families is generally attributed to segmental duplication and tandem duplication [36]. The duplication of *CsSPXs* was revealed by analyzing the syntenic regions through the MCscanX software. The cucumber genome was found to encompass 231 segmental duplication blocks and 1468 tandem duplication gene pairs (Table S2). Our synteny analysis identified four segmental duplication gene pairs within the cucumber *SPX* gene family (*CsSPX1*/*CsSPX2*; *CsSPX1*/*CsSPX5*; *CsPHO1.4*/*CsPHO1.6*; *CsSPX-MFS1*/*CsSPX-MFS2*), while no instances of tandem duplication gene pairs were detected (Fig. 4A; Table S2).

The syntenic maps of tomato, cucumber and *Arabidopsis* were compared to elucidate the phylogenetic mechanism of the cucumber *SPX* family (Fig. 4B; Table S3). Seven *CsSPX* genes in cucumber showed collinearity with *SPX* genes in tomato and *Arabidopsis*. Our findings suggested that the *CsPHO1.6* gene was linked to more than two syntenic gene pairs between cucumber and *Arabidopsis*. Furthermore, *CsSPX4* and *CsSPX*-*MFS1* genes were also associated with two syntenic gene pairs between cucumber and tomato, indicating that these *SPX* genes might play a pivotal role in the evolutionary process. Additionally, we discovered that specific collinear pairs were present between cucumber and both *Arabidopsis* and tomato, as exemplified by *CsSPX4*, *CsPHO1* and CsSPX-MFS1 (Fig. 4B; Table S3). This highlighted that these orthologous pairs could have already existed prior to the ancestral divergence.

### Analysis of *cis*-elements in*CsSPX* genes

**Fig. 5:**
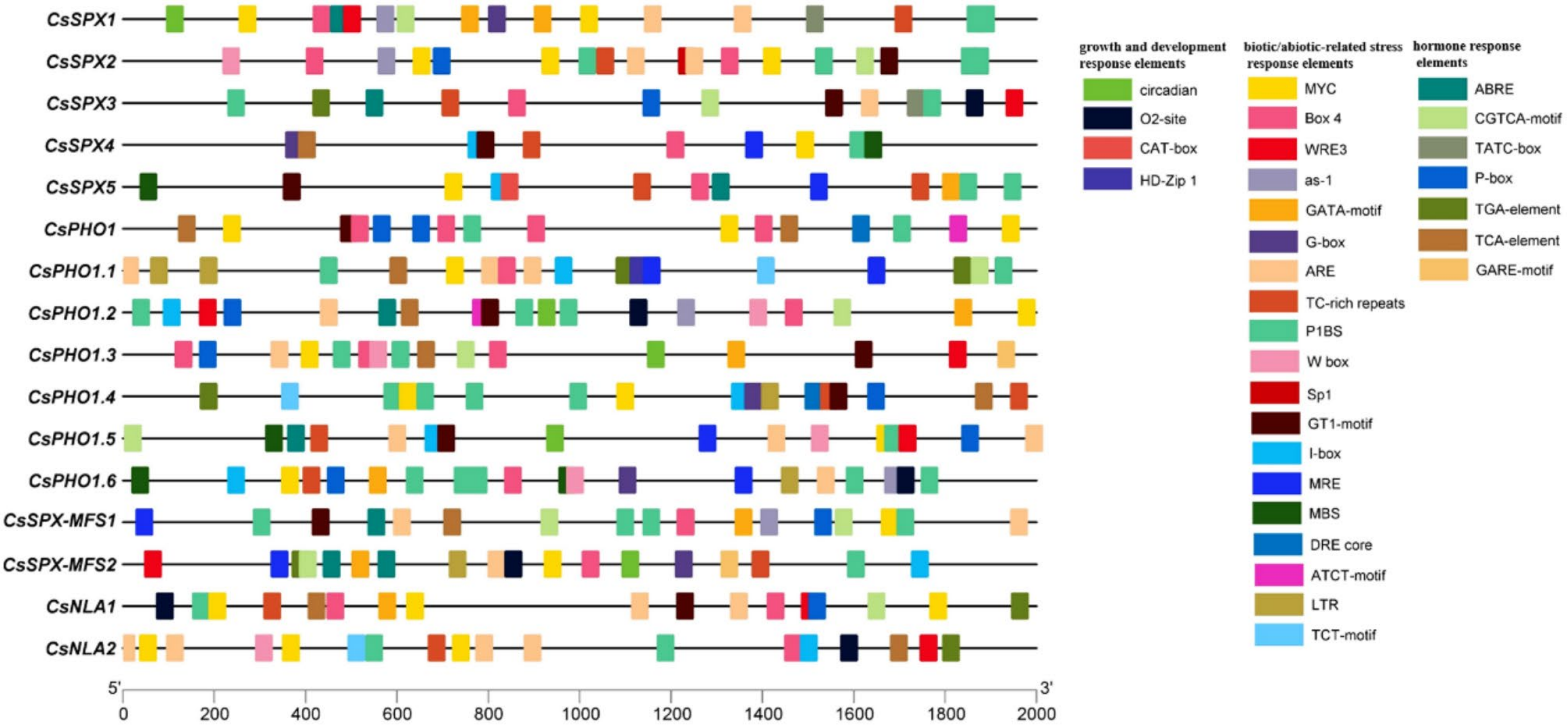
Depiction of *cis*-elements in *CsSPX* gene promoters. They were grouped into three main categories: biotic and abiotic stresse responses, growth and developmental responses and hormonal responses

**Fig. 6:**
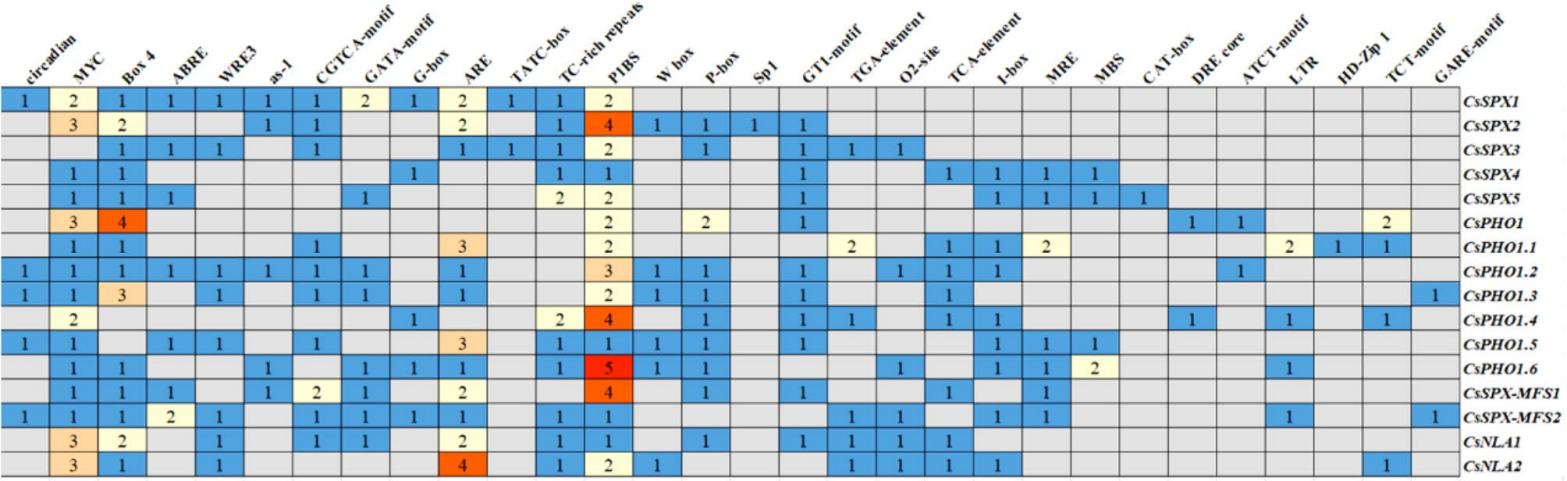
Statistical information on *cis*-elements in *CsSPXs*. The numerical values within the boxes correspond to the count of each type of *cis*-element

*Cis*-elements embedded within gene promoters are important for regulating gene expression. We utilized PlantCARE and PlantPAN 3.0 software to predict the *cis*-elements present in the 2-kb upstream region of the transcription start site of the *CsSPX* genes and classified them into three types: biotic/abiotic-related stress response elements, growth and development response elements, and hormone response elements (Figs. 5 and 6; Table S4). The stress-related elements encompassed light response components, such as the G-box, ATCT-motif, GT1-motif, TCT-motif, I-box, Box4, GATA-motif, ARE and Sp1, as well as low temperature response element LTR, drought response elements including MBS, MYC, DRE core and TC-rich repeats, and damage response elements WRE3. Our findings indicated that the promoters of several *CsSPX* genes contained an abundance of W box elements, which implied that these genes might be targeted by WRKY transcription factors. Promoters also frequently contained elements associated with developmental processes, including the O2-site, circadian, CAT-box and HD-Zip 1. Furthermore, seven hormone elements relating to phytohormones were identified (Figs. 5 and 6; Table S4). S*PX* genes have crucial functions in maintaining Pi homeostasis, and their promoters contain P1BS elements (PHR1 binding site) that are vital for responding to Pi starvation. The promoters of all *CsSPX* genes exhibited a pronounced enrichment of P1BS elements, with multiple copies of such elements present, suggesting that CsPHR1 might regulate these genes under Pi-deficient conditions.

### Predicting the interaction network of CsSPX proteins

**Fig. 7:**
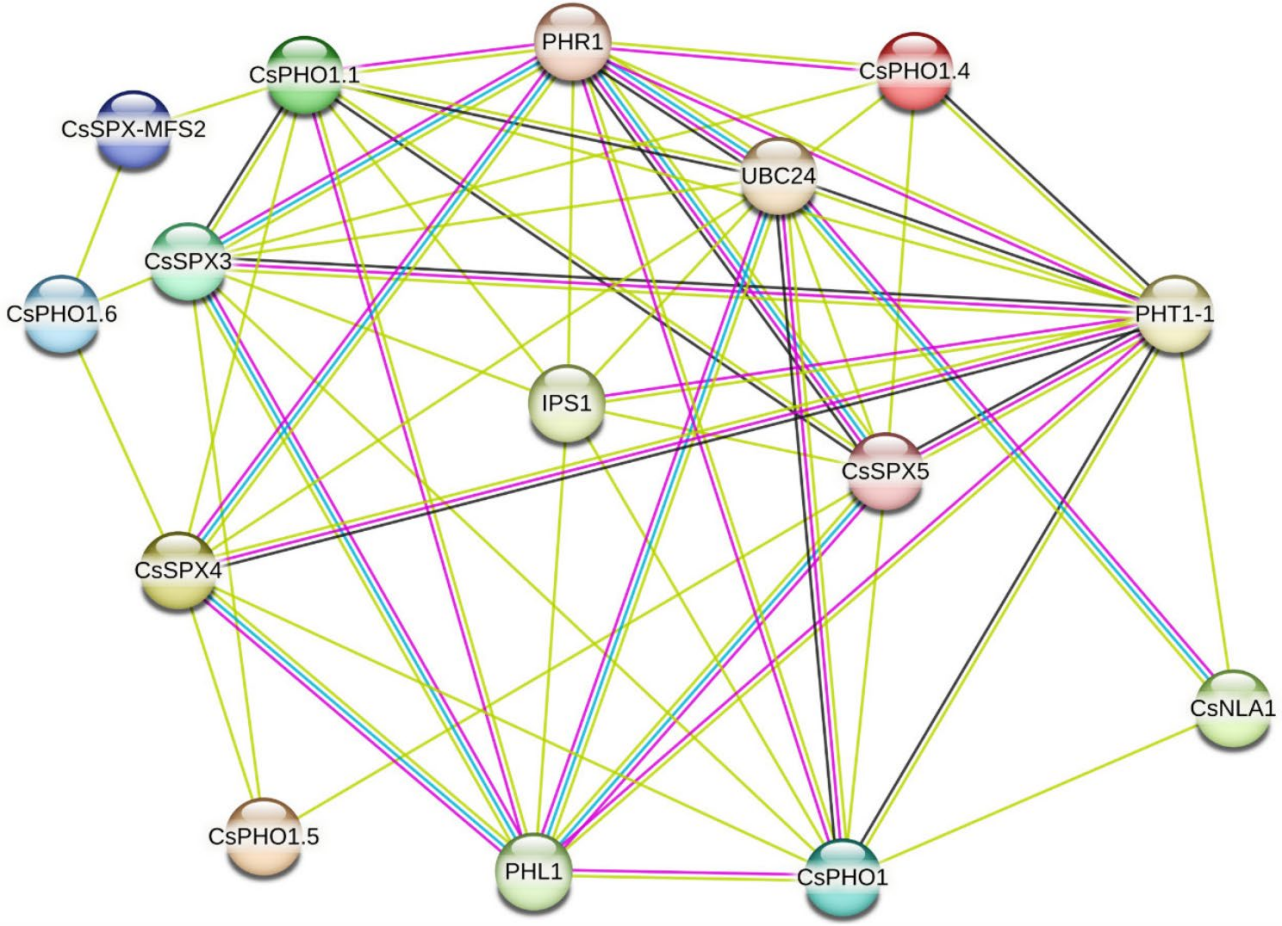
CsSPX protein interaction network. The STRING online software was chosen to build the network. The various lines of different colours represented diverse types of evidence used to predict the interaction network

Protein interaction network analysis has proven to be an effective method for studying gene function. Protein interaction networks were predicted through a STRING online software query of 16 CsSPX protein sequences. The analysis of the CsSPX protein interaction network revealed that several CsSPX proteins interacted with each other. For instance, CsPHO1.1 was predicted to engage in potential interactions with CsSPX3, CsSPX-MFS2 and CsSPX5 as illustrated in Fig. 7. The PHR proteins are recognized as essential transcription factors in the plant phosphorus regulatory network, playing a pivotal role in modulating signaling pathways during Pi starvation. PHR1 or PHL1 typically interacts with proteins that have SPX domains in a Pi-dependent way to control transcription. The observed interactions between several CsSPX proteins and Pi starvation response (PHR1 and PHL1) proteins were discovered, further emphasising the significance of SPXs in sustaining phosphorus homeostasis. We also found that IPS1 interacted with certain CsSPX proteins. Additionally, inorganic phosphate transporter 1–1 (PHT1-1), as a high-affinity transporter for external inorganic phosphate, had also been identified to interact with numerous CsSPX proteins (Fig. 7). Moreover, UBC24, a ubiquitin-conjugating enzyme E2 24, which negatively regulates the protein abundance of PHF1 and PHT1s under Pi sufficient conditions by facilitating the degradation of PHT1 proteins at the endomembrane, was also identified within the interaction network. The protein association network delineated in this study provided a valuable resource for future investigations into the molecular mechanisms underlying phosphorus regulation in plants.

### CsSPXs interact with CsPHR1

**Fig. 8:**
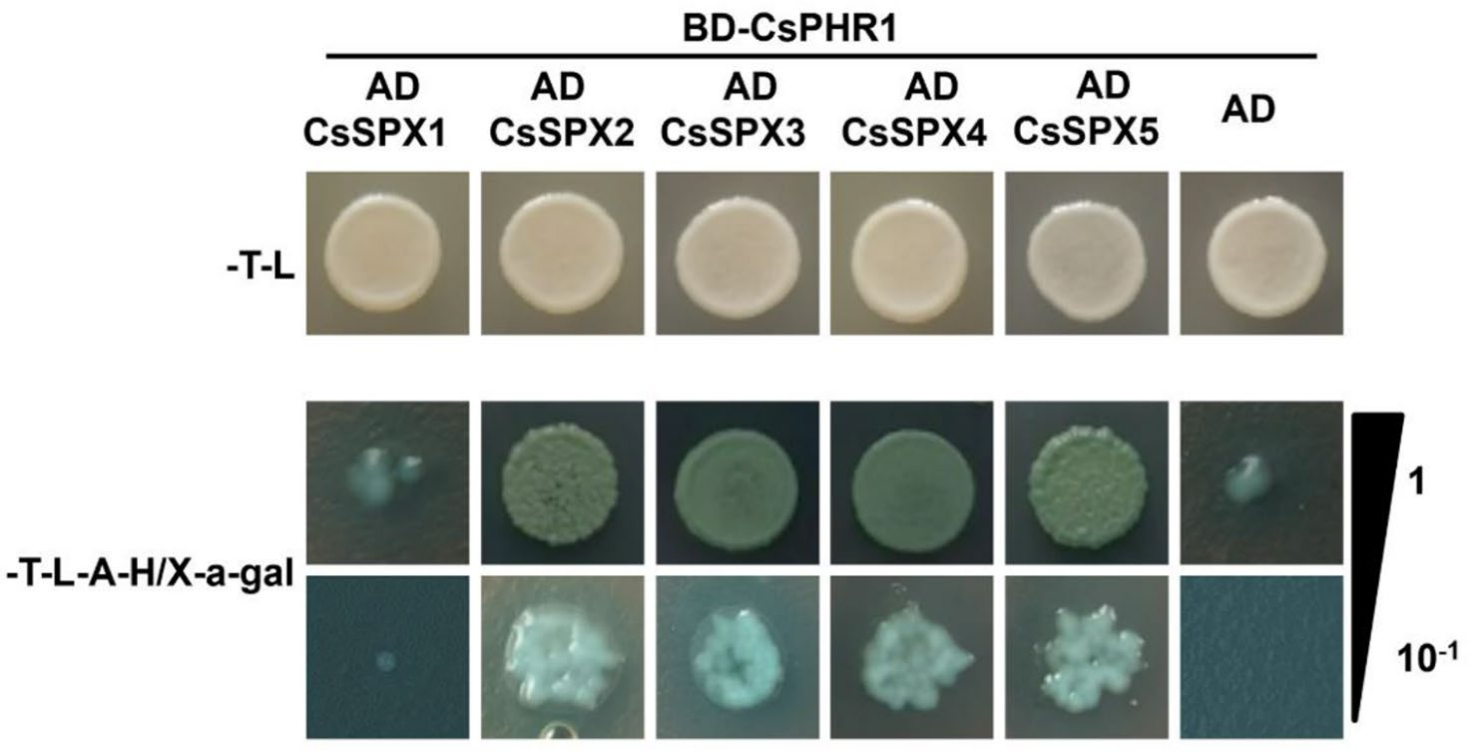
Yeast two-hybrid assays validation of CsPHR1 interactions with CsSPX proteins

The protein interaction network showed that CsPHR1 interacted with numerous CsSPX proteins. Yeast two-hybrid experiments were conducted to ascertain whether CsPHR1 interacts with CsSPXs. The BD-CsPHR1 bait construct was co-transformed with each of the recombinant plasmids (AD-CsSPX1 through AD-CsSPX5) into yeast cells. The growth of yeast cells was then observed on SD/-Trp-Leu-His-Ade medium with X-a-gal. Figure 8 illustrated that CsPHR1 interacted with CsSPX2, CsSPX3, CsSPX4 and CsSPX5, while not interacting with CsSPX1.

### Expression patterns of 16 *CsSPX* genes in different tissues of cucumber

**Fig. 9:**
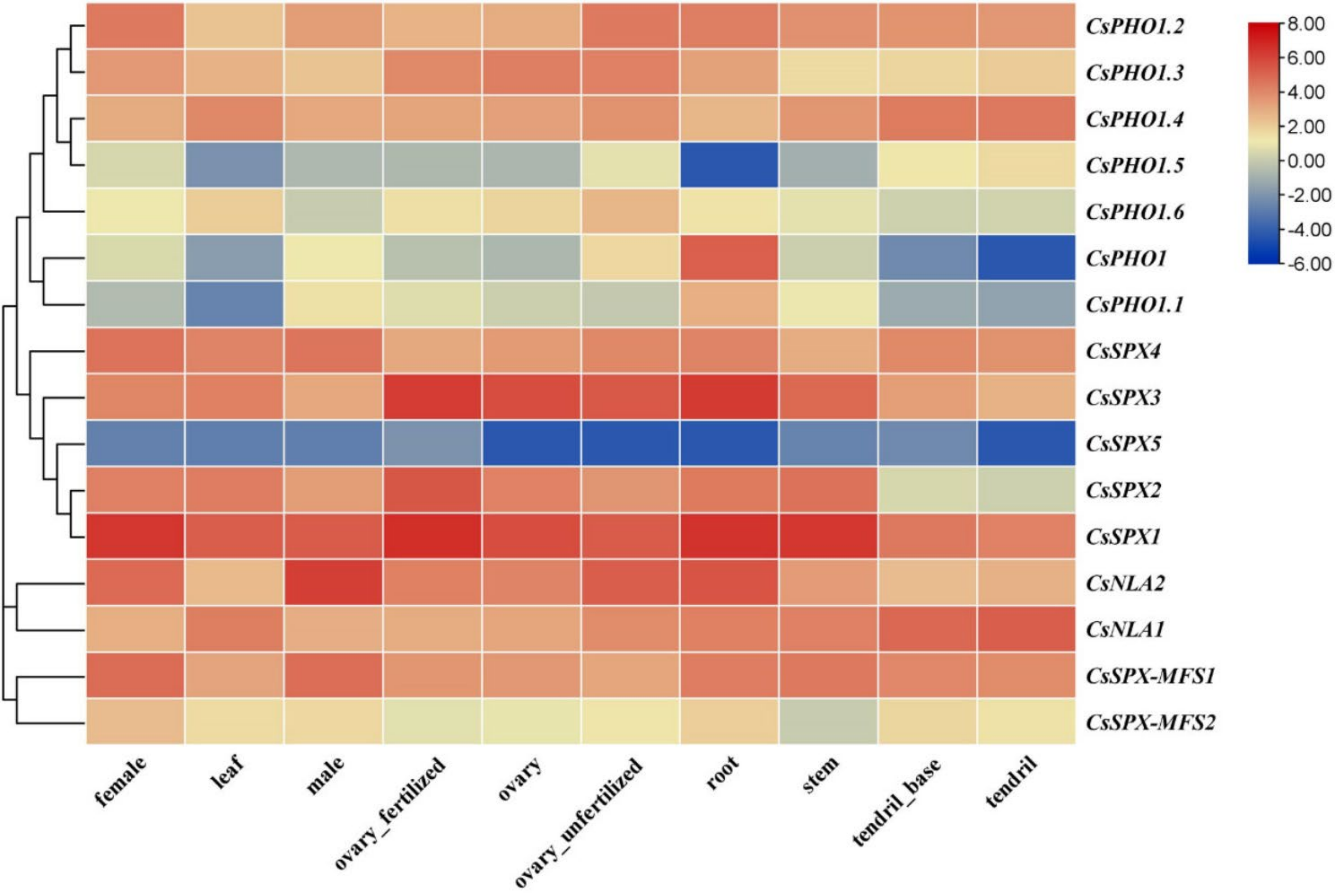
Tissue-specific expression of *CsSPX* genes. An analysis of the expression levels of the *CsSPX* genes in nine different tissues of cucumber 9930, utilizing publicly available transcriptomic data

To examine the expression patterns of the 16 *CsSPX* genes in various cucumber tissues, we analyzed their expression levels based on publicly available transcriptomic data from different tissues of cucumber (female, leaf, male, ovary_fertilized, ovary, ovary_unfertilized, root, stem, tendril_base and tendril) [37]. The results revealed that four of the five genes in the *SPX* subfamily were expressed in all tissues (FPKM > 0), except for *CsSPX5* (Fig. 9; Table S5). Notably, the *CsPHO1* and *CsPHO1.1* genes, which belong to the *SPX-EXS* subfamily, displayed markedly higher expression levels in root tissues, suggesting their vital roles in regulating phosphorus homeostasis and translocation in roots. The *CsSPX-MFS1* and *CsSPX-MFS2* genes were also expressed in all tissues, but preferentially in female and roots. The *CsNLA1* of the *SPX-RING* subfamily exhibited significantly higher expression levels in tendrils than in other tissues, while *CsNLA2* was highly expressed in female and male, indicating potential key roles in phosphorus regulatory network in floral tissues (Fig. 9; Table S5). To further validate the reliability of the transcriptomic data, we conducted an independent assessment of the 16 *CsSPX* gene expression across a range of cucumber tissues, including roots, stems, leaves, male flowers, ovaries and tendrils, employing qRT-PCR. The expression profiles obtained from this analysis corroborated the patterns observed in the transcriptomic dataset, as depicted in Fig. S3.

### Assessment of differential cucumber cultivar responses to low pi stress

**Fig. 10:**
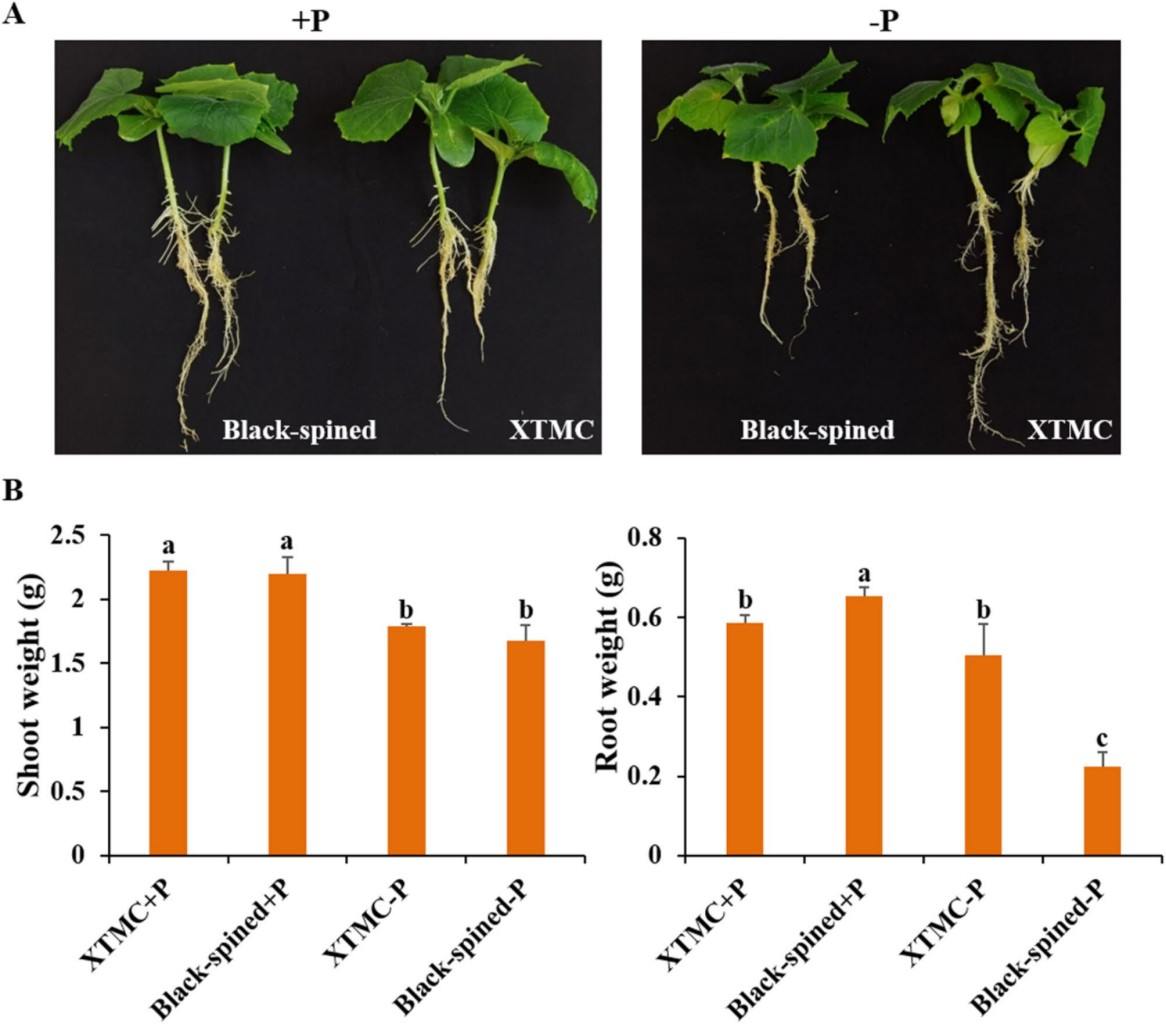
Phenotypic disparities of Xintaimici and black-spined cucumber cultivars subjected to low Pi stress. Phenotypic observations were made on Xintaimici and black-spined cucumber after 10 days of normal and low Pi stress growth conditions

The hydroponic experiments revealed that under normal Pi conditions, there was no significant growth disparity between the Xintaimici and black-spined cucumber cultivars. However, the shoot weight of both cucumber cultivars decreased under low Pi stress, but black-spined cucumber was more sensitive to low Pi stress, with significant changes in root weight in black-spined cucumber, but not in Xintaimici (Fig. 10). Some *CsSPX* subfamily genes were highly induced by low Pi stress, with CsSPX1 and CsSPX2 being particularly responsive (Fig. S4). Notably, we found that the expression levels of *CsSPX1*, *CsSPX2* and *CsPHR1*in Xintaimici cucumber were significantly higher than those in black-spined cucumber under low Pi stress (Fig. S5). This differential expression pattern underscored the pivotal role of *CsSPX1* and *CsSPX2* in the acclimation to phosphorus deficiency, potentially contributing to the superior Pi stress tolerance exhibited by the Xintaimici cucumber.

## Discussion

Phosphorus (Pi) is a critical mineral element that is indispensable for plant growth and development, playing a pivotal role in many metabolic pathways of plants, such as enzymatic reaction, sugar metabolism, photosynthesis and energy metabolism [2]. Physiological and metabolic processes in plants responding to phosphorus deficiency rely on a sophisticated and intricate regulatory network of phosphorus signaling within their systems [38]. With the advancements in molecular biology research techniques, a greater number of transcription factors and phosphorus starvation-responsive genes involved in plant phosphorus signaling regulatory pathways have been identified, significantly enhancing our understanding of the phosphorus signaling network and their roles in plant adaptation to Pi limitation. Studies have shown that WRKY transcription factors have been implicated in the regulation of Pi-starvation responses [12]. In addition, one of the well-studied transcription factors is PHR1 (Phosphate Starvation Response 1), which plays a pivotal role in the regulation of Pi homeostasis in *Arabidopsis thaliana*. PHR1 binds to the promoters of target genes, such as those encoding for high-affinity phosphate transporters, and regulates their expression in response to Pi availability [23]. Another key player in Pi signaling is the *SPX* domain-containing family of proteins, which have been the focus of recent investigations into the modulation of Pi signaling in model plants such as *Arabidopsis*, maize, wheat and rice. Despite these advances, the functional and evolutionary studies of *CsSPXs* genes in cucumber remain underexplored. In this study, 16 *CsSPX* genes were identified, which were classified into four distinct subfamilies based on their structural and sequence homology (Fig. 2; Table 1). Within multi-genic families, gene structure and motif patterns can provide valuable insights into the genetic relationships and potential functional redundancies among family members [39, 40]. Figure 2 illustrated that SPXs exhibited a close similarity in gene structure and motif composition within the same clade. Genes with analogous structures and conserved motifs typically share comparable functions. Cucumber SPX proteins were categorized into several functional clades in *Arabidopsis*, providing valuable insights into gene function of *CsSPX*. The construction of a comprehensive phylogenetic tree, encompassing 147 SPX proteins from six species, allowed us to infer the evolutionary relationships and diversification of *CsSPX* genes in cucumber relative to other plant species (Fig. 3). Compared to the *SPX* family genes present in wheat, maize, and rice, the *SPX* family genes found in cucumber were distinctly separated into different evolutionary branches. Furthermore, the evolutionary relationships between members of the *SPX*-*EXS* and *SPX*-*MFS* subfamilies significantly differed between species, implying that the roles of these two subfamily proteins may differ across different plants.

As in other species, the promoters of the *CsSPX* gene were found to harbor a diverse array of *cis*-elements associated with biotic and abiotic-related responses, as well as hormone signaling [32, 33]. Studies have shown that *SPX* family genes can be regulated by WRKY transcription factors. For instance, the *AtPHO1* gene can be regulated by WRKY6 and WRKY42 through targeting the W-box element of its promoter [12]. In our analysis, six *CsSPX* genes were identified to contain W-box elements within their promoter regions, suggesting possible regulation by WRKY transcription factors (Figs. 5 and 6). The P1BS (GNATATNC) element, known for its involvement in the response to Pi deficiency, is enriched in the promoters of many *PSR* genes [38, 41, 42]. For instance, the P1BS elements are prevalent in the promoter regions of 18 *ZmSPX* genes and 31 *BnaSPX* genes [14, 32]. In this study, all *CsSPX* genes contained varying amounts of P1BS elements in their promoter regions (Figs. 5 and 6), indicating a conserved regulatory mechanism in response to Pi deficiency in cucumber. The activity of a promoter region is directly influenced by the position, quantity, and flanking sequence of all elements. The diversity of these elements implied a complex regulatory landscape for *CsSPXs* genes, potentially involving multiple associated transcription factors.

The expression analysis of *CsSPXs* genes under varied durations of low Pi treatment indicated that these genes exhibit diverse responses to Pi stress. Notably, *CsSPX1*, *CsSPX2* and *CsPHR1* exhibited diverse expression patterns in Xintaimici and black-spined cucumber, leading to dissimilar alterations in root weight (Fig. 10; Fig. S4; Fig. S5). Several studies have demonstrated that under low Pi conditions, certain *SPX* subfamily genes are up-regulated, such as *AtSPX1* and *AtSPX2* [23]. In our study, the *CsSPX* subfamily gene members were markedly induced when cucumber lacked Pi, which was consistent with the results of previous studies [20, 43].

Utilizing the STRING online software, we predicted the protein interaction networks for the 16 CsSPX protein sequences, revealing potential interactions between several CsSPX proteins and CsPHR1. AtPHR1 is known to play a role in Pi homeostasis by interacting with SPX domain-containing proteins under low Pi conditions [23]. The Y2H assay results demonstrated that CsSPX2, CsSPX3, CsSPX4 and CsSPX5 exhibited interaction with CsPHR1, whereas CsSPX1 displayed no interaction with CsPHR1, similar to that found in maize and rice. This indicated that the regulatory network for responding to Pi stress in cucumber was relatively conserved, providing a foundation for further functional studies of these interactions and their implications for phosphorus metabolism in plants.

The differential expression of *CsSPX* genes in response to Pi stress and their interactions with CsPHR1 underscored the complexity of the phosphorus signaling network in cucumber. These findings not only contribute to our understanding of how cucumber adapts to Pi limitation but also have implications for crop improvement. By identifying key regulatory genes and their interactions, we can potentially manipulate the expression of these genes to enhance phosphorus use efficiency and improve crop yield under Pi-deficient conditions. Future research should focus on functional validation of these candidate genes and exploring their roles in the broader context of plant phosphorus nutrition. Additionally, comparative studies across a wider range of plant species will provide further insights into the evolution and diversification of phosphorus signaling pathways, which could inform strategies for the development of plants with improved tolerance to Pi deficiency.

## Conclusions

In this study, a total of 16 *CsSPX* genes were identified in the cucumber genome and systematically classified into four distinct subfamilies. A detailed analysis of gene duplications, phylogenetic relationships, *cis*-elements, gene structures and conserved motifs was carried out to comprehensively describe the biological characteristics of these *CsSPX* genes. The expression analysis of the 16 *CsSPXs* during various stages of Pi stress revealed that *CsSPXs* have crucial involvement in the response to Pi starvation. Particularly, *CsSPX1* and *CsSPX2* exhibited a pronounced response to low Pi stress conditions. Certain SPXs might have functions under Pi starvation through interaction with PHR1. In essence, this study has significantly advanced our understanding of the cucumber *SPX* gene family, offering a substantial foundation for forthcoming biological explorations into the realm of *SPX* genes.

## Methods

### Identifying *CsSPX* Genes in cucumber

Using 20 *Arabidopsis* SPX proteins as query sequences, Blastp search was performed on cucumber genome database to screen for members of the *CsSPX* gene family. Subsequently, the Pfam database (http://pfam.xfam.org/) was utilized to further refine the search for *CsSPX* genes by employing the Hidden Markov Model (HMM) SPX domain (PF03105). Verification of all candidate genes was carried out by employing the Pfam and SMART (http://smart.embl-heidelberg.de) tools. Through this systematic approach, 16 *CsSPX* genes were meticulously selected from the cucumber genome and designated based on their evolutionary relationship to *AtSPXs*.

### Alignment of multiple sequences and analysis of their phylogeny

Multiple alignments of SPX proteins from 20 AtSPXs, 15 OsSPXs, 33 ZmSPXs, 18 SlSPXs, 16 CsSPXs and 46 TaSPXs were conducted using CLUSTALW to elucidate their evolutionary relationships across different species. The obtained results were used to develop a phylogenetic tree through the neighbour-joining (NJ) method in MEGA 7 [44], with support from 1000 bootstrap replicates to ascertain the robustness of the branching patterns. The phylogenetic tree was subsequently visualized and refined using the EvolView tool (http://www.evolgenius.info) [45]. The tree was further categorized into four distinct subfamilies, aligning with the known characteristics of the *SPX* gene family, to provide a clearer understanding of the functional and evolutionary diversification within this protein family.

### Investigation of gene structures and conserved motifs within *CsSPX* and *AtSPX* family members

We extracted the DNA and cDNA sequences of all *CsSPX* and *AtSPX* genes from the genomes of cucumber and *Arabidopsis*, respectively.The Gene Structure Display Server (GSDS: http://gsds.cbi.pku.edu.cn/) [46] was employed to analyze and illustrate the architectural composition of these genes. Additionally, we identified conserved motifs in all CsSPX and AtSPX amino acid sequences by the MEME tool (http://meme-suite.org/index.html). TBtools was used to visualize the results.

### Physicochemical properties and chromosome location analysis

The amino acid lengths, molecular weights and isoelectric points (pI) of the 16 CsSPX protein sequences were predicted using the ExPASy online website (http://web.expasy.org/protparam/). The chromosomal allocation of all *CsSPXs* genes within the cucumber genome was meticulously mapped using TBtools [47], utilizing physical location data extracted from the cucumber genome database.

### Gene duplication analysis

The gene duplication events were analyzed using the Multiple Collinearity Scan toolkit (MCScanX) with the default parameters [48]. Syntenic analysis maps were generated using TBtools to display the syntenic relationships of orthologous *SPXs* in cucumber, tomato and *Arabidopsis*.

### Identifying *cis*-elements in the promoters of *CsSPX* genes

Sequences of 2 kb upstream of the transcription start site for the 16 *CsSPX* genes were obtained from the cucumber genome database with the TBtools. Next, we uploaded the entire set of sequences to the PlantCARE (http://bioinformatics.psb.ugent.be/webtools/plantcare/html/) and PlantPan3 databases (http://plantpan.itps.ncku.edu.tw/plantpan4/promoter_analysis.php) for the purposes of identifying and characterizing *cis*-elements [49]. The collation and visualisation of the results was then performed by TBtools.

### Creating protein association networks with STRING

We uploaded the amino acid sequences of the 16 CsSPX to the online STRING software (version 11.5; http://string-db.org) and selected “*Arabidopsis thaliana*” as the reference organism. We constructed the network by selecting proteins with the highest interaction scores after conducting a BLAST analysis. Proteins lacking interactions with other entities were excluded from the network. We manually transferred the functional annotations from the BLAST results. The names of PHR1, IPS1, PHL1, PHT1-1 and UBC24 proteins were provided automatically by the system, streamlining the process of network annotation and analysis.

### Yeast two-hybrid (Y2H) assays

Recombinant plasmids AD-CsSPX1, AD-CsSPX2, AD-CsSPX3, AD-CsSPX4, AD-CsSPX5 and BD-CsPHR1 were prepared for Y2H assays by cloning the full-length *CsPHR1* coding sequence (CDS) into the bait vector pGBKT7 and the CDSs of *CsSPX1*, *CsSPX2*, *CsSPX3*, *CsSPX4* and *CsSPX5* into the prey vector pGADT7. Y2H assays were performed in accordance with the manufacturer’s instructions with yeast strain AH109 (Clontech). Yeast cells were grown at 28 °C on synthetic dropout medium lacking Trp and Leu (SD/-Trp-Leu). To screen interactions, colonies were transferred to medium lacking Trp, Leu, His and adenine (SD/-Trp-Leu-His-Ade) containing X-a-gal. A negative control experiment was carried out using empty vector pGADT7 [50]. Table S6 provided the primers that were used in this study.

### Plant growth conditions and low pi stress treatments

The black-spined cucumber utilized in this study, characterized by its black spines, is preserved by our laboratory. Seeds of Xintaimici and black-spined cucumber cultivars were germinated on moist filter paper in an incubator at 28 °C for 1 day. Then, the germinated seeds were sown into the substrate mixture, and after 10 days, the seedlings were gently washed to remove any residual soil, and then transferred to nutrient solution containing whole nutrients for hydroponics. Following two days of equilibration, the nutrient solution was exchanged with the full nutrient treatment containing KH_2_PO_4_ 1 mM (+ P) and the full nutrient treatment containing KH_2_PO_4_ 10 µM low Pi treatment (-P), respectively. Root samples were subsequently collected at 0 h, 3 h, 6 h, 12 h, 24 h and 48 h and immediately frozen in liquid nitrogen for semi-quantitative analysis.

### RNA extraction and qRT-PCR analysis

Total RNA was isolated from the designated samples using a plant RNA extraction kit (TianGen, Beijing, China). The isolated RNA was then reverse transcribed into cDNA utilizing the PrimeScript^®^ 1st Strand cDNA Synthesis Kit (Takara, Japan). The qRT-PCR was executed according to the previously outlined method [50]. The expression levels were normalized against the internal control, the cucumber *β-actin* gene. Each analysis was conducted with three biological replicates to ensure experimental reliability.

### Electronic supplementary material

Below is the link to the electronic supplementary material.

Supplementary Material 1

Supplementary Material 2

Supplementary Material 3

Supplementary Material 4

Supplementary Material 5

Supplementary Material 6

Supplementary Material 7

## Data Availability

The data that support the results are included within the article and its additional files. Other relevant materials are available from the corresponding authors on reasonable request.
