# Supplementary material for: Mining key genes associated with phosphorus deficiency through genome-wide identification and characterization of cucumber SPX family genes
Source: BMC Plant Biol. 2024 Jul 24;24:699. doi: 10.1186/s12870-024-05436-3 (PMC11267760; doi:10.1186/s12870-024-05436-3)
Supplement: Supplementary file 7 — Supplementary Material 7 [file 12870_2024_5436_MOESM7_ESM.pdf]

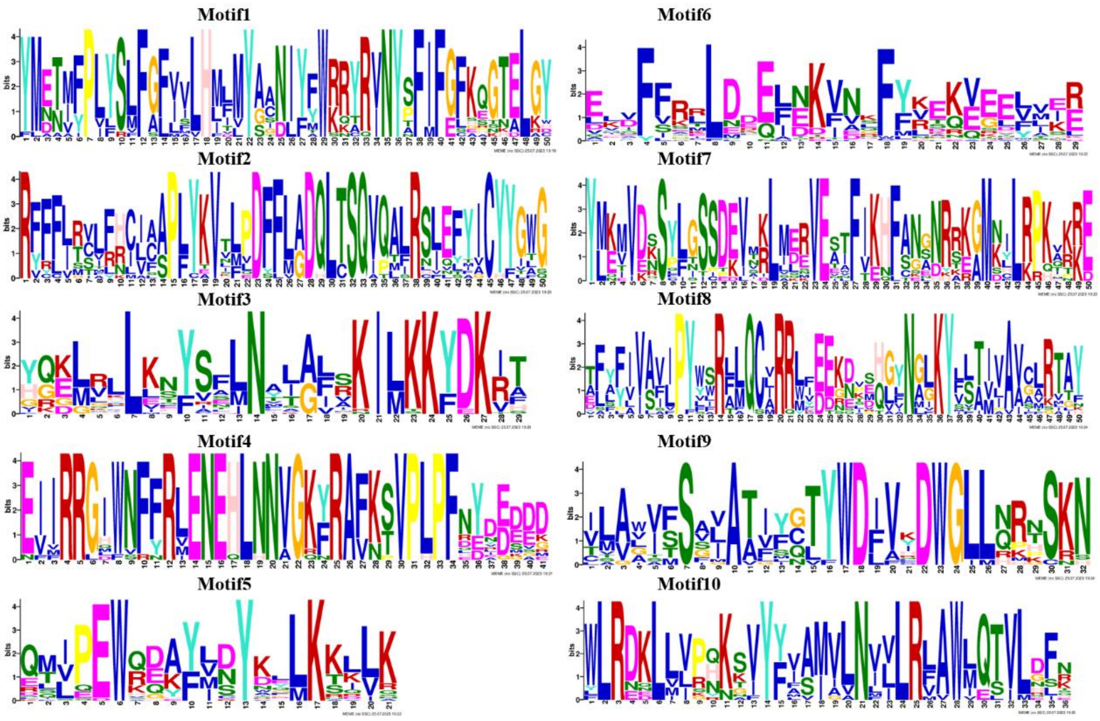

Fig. S1. The logos for the 10 conserved motifs of CsSPX proteins derived from the MEME Suite.

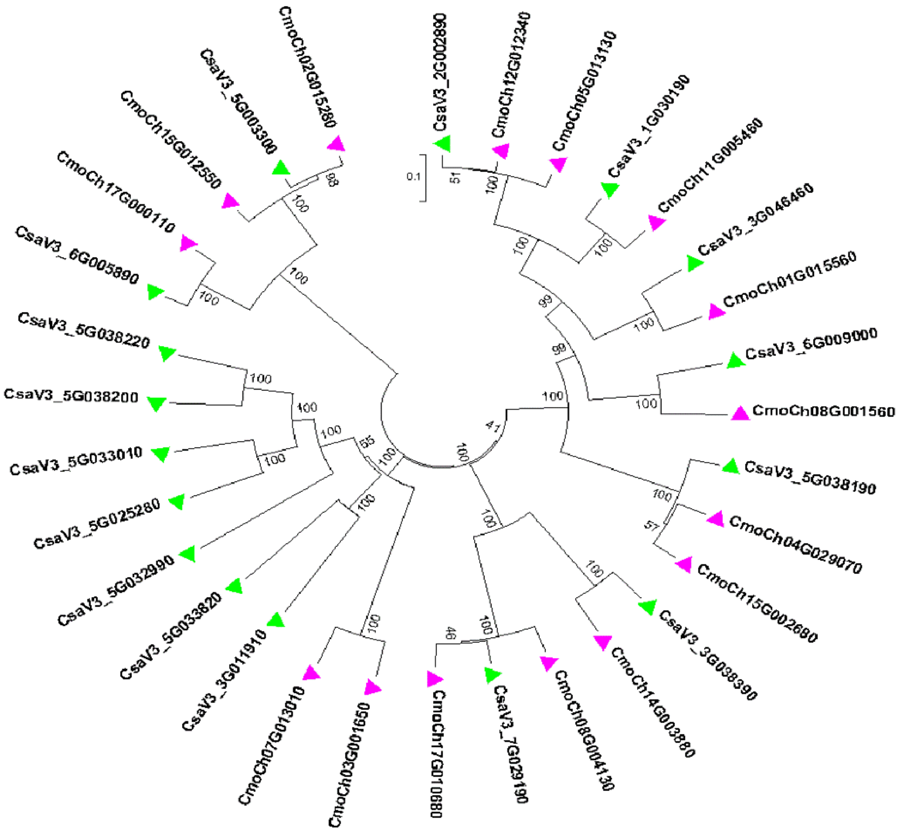

Fig. S2. Phylogenetic analysis of SPX proteins in *Cucumis sativus* and *Cucurbita moschata*.

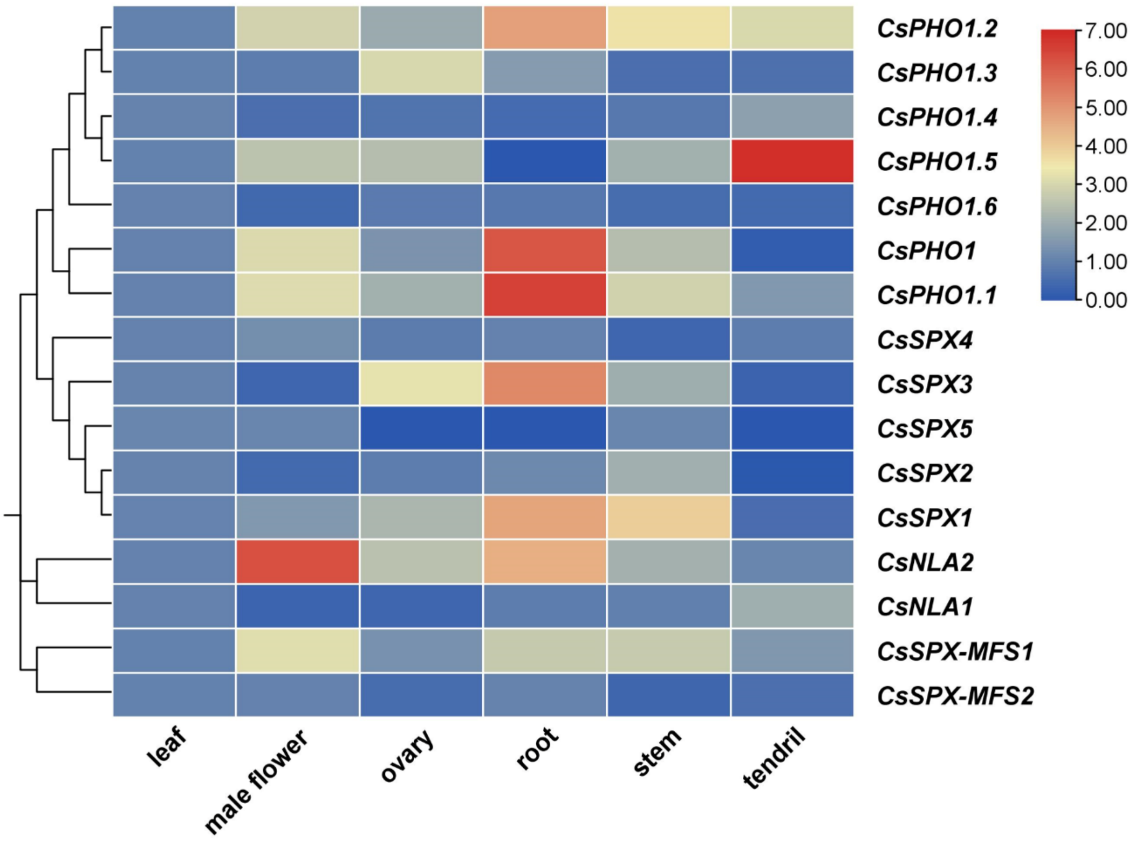

Fig. S3. Validate the expression levels of the 16 *CsSPX* genes in different tissues of cucumber using qRT-PCR. The expression levels were normalized against the internal control, the cucumber *β-actin* gene. Each experiment was conducted with three biological replicates to ensure the reliability of the results.

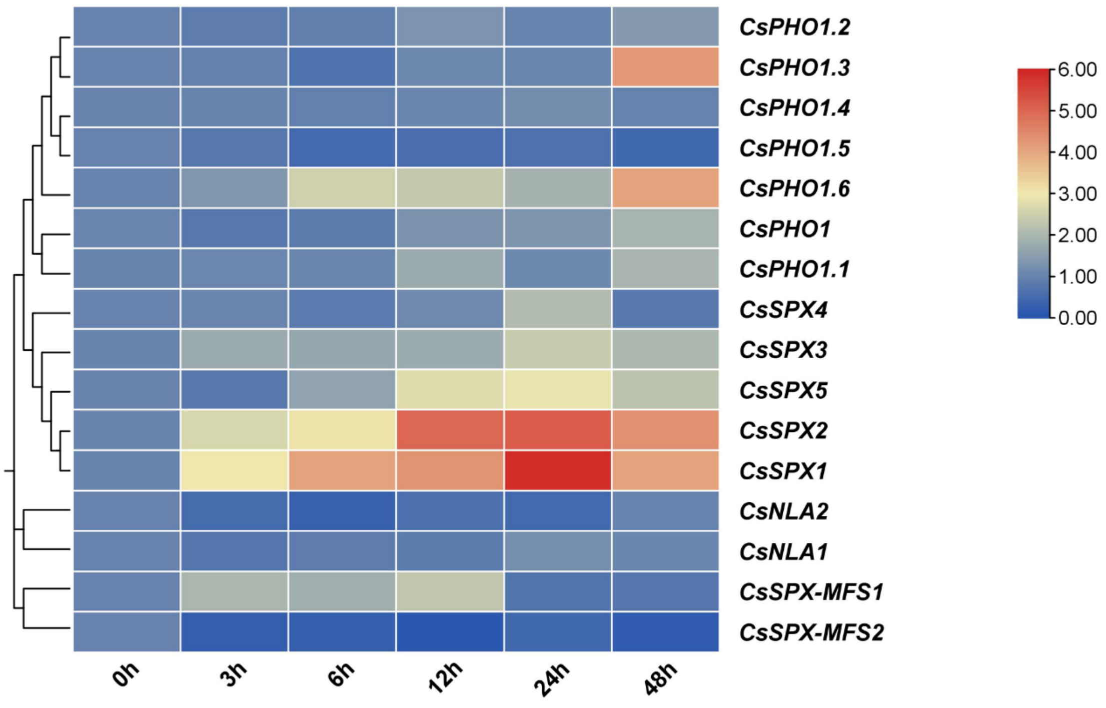

Fig. S4. Expression analysis of 16 *CsSPX* genes in cucumber root at different times of low Pi treatment. The expression levels were normalized against the internal control, the cucumber *β-actin* gene. Each experiment was conducted with three biological replicates to ensure the reliability of the results.

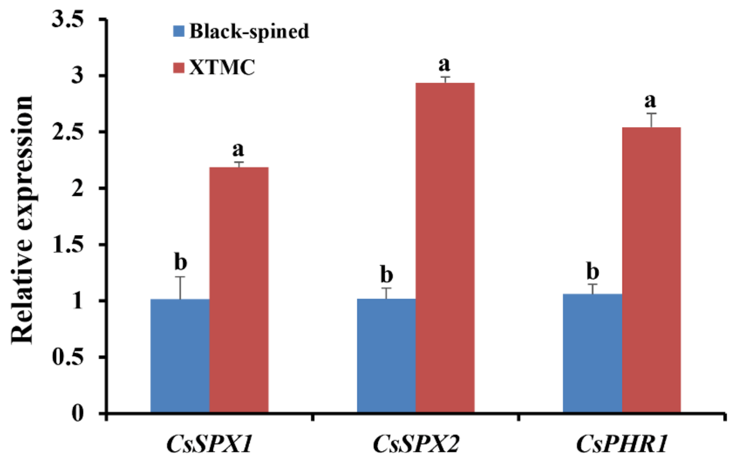

Fig. S5. Expression levels of *CsSPX1*, *CsSPX2* and *CsPHR1* in the roots of Xintaimici (XTMC) and black-spined cucumber after 6 hours of low Pi treatment. The expression

levels were normalized against the internal control, the cucumber *β-actin* gene. Each experiment was conducted with three biological replicates to ensure the reliability of the results. Lowercase letters represented significant differences ($P < 0.05$).
